# High-resolution characterization of sequence signatures due to non-random cleavage of cell-free DNA

**DOI:** 10.1186/s12920-015-0107-z

**Published:** 2015-06-17

**Authors:** Dineika Chandrananda, Natalie P. Thorne, Melanie Bahlo

**Affiliations:** Bioinformatics Division, The Walter and Eliza Hall Institute of Medical Research, Parkville, Melbourne, VIC 3052 Australia; Department of Medical Biology, University of Melbourne, Melbourne, VIC 3010 Australia; Department of Mathematics and Statistics, University of Melbourne, Melbourne, VIC 3010 Australia

**Keywords:** Cell-free DNA, extracellular DNA, biomarkers, fragment lengths, fragmentation motifs, nucleosomes, higher-order chromatin packaging, apoptosis, necrosis

## Abstract

**Background:**

High-throughput sequencing of cell-free DNA fragments found in human plasma has been used to non-invasively detect fetal aneuploidy, monitor organ transplants and investigate tumor DNA. However, many biological properties of this extracellular genetic material remain unknown. Research that further characterizes circulating DNA could substantially increase its diagnostic value by allowing the application of more sophisticated bioinformatics tools that lead to an improved signal to noise ratio in the sequencing data.

**Methods:**

In this study, we investigate various features of cell-free DNA in plasma using deep-sequencing data from two pregnant women (>70X, >50X) and compare them with matched cellular DNA. We utilize a descriptive approach to examine how the biological cleavage of cell-free DNA affects different sequence signatures such as fragment lengths, sequence motifs at fragment ends and the distribution of cleavage sites along the genome.

**Results:**

We show that the size distributions of these cell-free DNA molecules are dependent on their autosomal and mitochondrial origin as well as the genomic location within chromosomes. DNA mapping to particular microsatellites and alpha repeat elements display unique size signatures. We show how cell-free fragments occur in clusters along the genome, localizing to nucleosomal arrays and are preferentially cleaved at linker regions by correlating the mapping locations of these fragments with ENCODE annotation of chromatin organization. Our work further demonstrates that cell-free autosomal DNA cleavage is sequence dependent. The region spanning up to 10 positions on either side of the DNA cleavage site show a consistent pattern of preference for specific nucleotides. This sequence motif is present in cleavage sites localized to nucleosomal cores and linker regions but is absent in nucleosome-free mitochondrial DNA.

**Conclusions:**

These background signals in cell-free DNA sequencing data stem from the non-random biological cleavage of these fragments. This sequence structure can be harnessed to improve bioinformatics algorithms, in particular for CNV and structural variant detection. Descriptive measures for cell-free DNA features developed here could also be used in biomarker analysis to monitor the changes that occur during different pathological conditions.

**Electronic supplementary material:**

The online version of this article (doi:10.1186/s12920-015-0107-z) contains supplementary material, which is available to authorized users.

## Background

The existence of cell-free DNA circulating in human plasma was discovered in 1948 [1] however, the study of this phenomenon was delayed in the following decades due to the lack of suitable laboratory techniques. In recent years, the use of methods such as PCR, and its more sophisticated derivatives, along with advances in next-generation sequencing have expanded our understanding of cell-free DNA, although many facets of its biology still remain unknown.

Current understanding of cell-free DNA encompasses that it exists as double-stranded molecules, which are biologically fragmented into both short (<1 Kb) and long segments (>10 Kb) [2, 3]. This disparity in size along with evidence from experimental and observational studies have led researchers to postulate apoptosis [4, 5], necrosis [2, 6] and active release [4, 7] as potential mechanisms that may produce extracellular DNA. The relative contributions of these three processes and how their contributions change in pathological conditions is still under investigation.

While first discovered in human plasma and serum, cell-free DNA has now been extracted from other body fluids such as urine [8, 9], cerebrospinal [10], synovial [11] and pleural [12] fluids. While a majority of cell-free DNA circulates as histone bound nucleosomal elements [13, 14], at least a portion of this DNA appears to be housed with lipoprotein virtosomes or held within membranous vesicles. These are believed to grant protection against further enzymatic degradation and recognition by immune cells that could trigger autoimmune responses. Such packaging is also hypothesized to play a part in the effective clearance of cell-free DNA [15]. Detailed information of the mechanisms associated with these processes is lacking and remain somewhat controversial.

With the evolution of next-generation sequencing (NGS), quantitative aspects of cell-free DNA as a potential non-invasive biomarker are being studied and utilized in diverse fields such as cancer genome scanning, prenatal testing, rheumatoid arthritis research as well as transplant rejection and dialysis monitoring [16–23]. Most published analyses on cell-free DNA appear to be clinically motivated. Therefore, research that further characterizes circulating DNA could substantially increase its diagnostic value by allowing the application of more sophisticated bioinformatics tools that lead to an improved signal to noise ratio.

There have been two studies of note in recent years that have documented the differences between cell-free and cellular DNA using sequencing data. A 2009 analysis by Beck et al. used low-coverage pyro-sequencing data (>0.001X) of serum cell-free DNA from 50 healthy individuals in comparison to cellular DNA matched to 4 of the subjects. They observed that essentially no functional genomic feature such as annotated genes was over-represented in cell-free DNA. The highest variations in coverage between the 50 subjects were documented in the representation of coding sequences (CDSs), untranslated regions (UTRs) and pseudogenes. This study also documented an over-representation of Alu elements in comparison to the genome as well as an under-representation of long interspersed nuclear elements L1 and L2 [24].

A 2012 study utilized SOLiD sequencing technology also revealed differences in repetitive-sequence representation between cell-free DNA from apoptotic human umbilical-vein endothelial cells and cellular DNA from the same living cells [25]. Alu repeats and certain satellite repeat subtypes were found to be over-represented, whereas L1 repeats were under-represented in the cell-free apoptotic DNA. L1 elements are mainly located in the transcriptionally inactive heterochromatin and Alu repeats localize to gene-rich euchromatin regions that have high rates of transcription. This disparity in repeat region presence in cell-free DNA fragments has also been documented by older studies [26, 27].

Other sequencing studies have examined cell-free DNA features such as fragment lengths with the most comprehensive analyses carried out on DNA originating from pregnancies [8, 28]. Circulating fetal DNA makes up 3 to 20 % of the total cell-free DNA in a pregnant woman’s plasma [29–31]. This percentage increases with gestation [32, 33] and has been shown to have an inverse relationship with maternal weight [33–35]. The primary origin of cell-free fetal DNA appears to be the syncytiotrophoblast of the developing embryo that makes up part of the placenta [36, 37]. Although there is evidence that both apoptosis and necrosis adds to the fetal DNA in the maternal circulation, there is no consensus on their relative contribution to the total pool of cell-free DNA [38]. The fetal fragments can be detected as early as the fourth week and reliably after the seventh week of gestation using PCR based methods [39, 40] and is cleared from the maternal blood within hours after childbirth [41, 42].

Studies have shown that the DNA molecules in maternal plasma have a size distribution that exhibits a number of peaks. The most prominent peak in their data was around 166 bp with the next occurring at 143 bp, followed by a series of smaller peaks at intervals of approximately 10 bp. The researchers also documented that a very small proportion of fragments exhibited lengths close to 350 bp. The peak at 166 bp is hypothesized to represent DNA that is wrapped around one nucleosomal unit while the peaks at 10 bp periodicity are related to the enzymatic cleavage of DNA wrapped around the histone core of each nucleosome. The studies also reported that fetal DNA was generally shorter than maternal DNA with fetal fragments showing a clear reduction of the 166 bp peak. Due to the selective nature of PCR used in the sequencing library preparation, these studies have been limited to investigation of lower molecular weight fragments (<1 Kb). The samples were sequenced at relatively low whole-genome coverage with cell-free DNA samples only averaging 10 million reads.

The advancement of bisulfite sequencing technology [43, 44] in the past few years has enabled the high-resolution interrogation of the epigenetic landscape of cell-free DNA. While the large background of maternal DNA makes it challenging to investigate fetal-specific methylation signals, recent studies report that placental tissue appeared to be generally hypo-methylated when compared with other somatic tissues and that fetal cell-free DNA has methylation profiles similar to the placental methylomes [45, 46]. Bisulphite analysis also shows that there are gestational age related epigenetic changes [47] and that longer fragments exhibit higher proportions of methylated CpG sites [48].

### Aims of study

In this work, we utilize previously published, matched cell-free and cellular DNA from two pregnant women, which have been sequenced to a very high depth resulting in some of the most high coverage datasets available in the cell-free DNA research field (>70X, >50X). The data was generated by Kitzman et al. [49] with the aim of assembling the fetal genome non-invasively along with genetic information from the mother and father. Here, we utilize this data in a descriptive approach to investigate the characteristics of cell-free DNA present in maternal plasma. Our aims are broadly two-fold: whilst expanding what is known about cell-free DNA in human plasma we endeavor to document cell-free DNA features that have the potential to be utilized as clinically actionable biomarkers either on their own or in conjunction with other known characteristics. The specific datasets used are ideal for our aims as they provide high-quality whole-genome information at a great sequencing depth along with the experimental set up of matched cellular and cell-free DNA.

This study examines the different signatures related to the enzymatic cleavage of cell-free DNA in an attempt to document the non-randomness associated with the process. The high density of sequencing reads has enabled a substantial extension of previous analyses into the fragment size distributions as we investigate the periodicity associated with the fragment lengths and how the lengths are dependent on the genomic location within chromosomes. We also show that the mapping locations of cell-free DNA fragments associate with arrays of nucleosomes on a genome-wide level by correlating them with nucleosome and open chromatin enrichment positions from ENCODE. The matched cell-free, cellular setup allows us to extend our previous work on determining the motif structure associated with cell-free DNA cleavage [50]. Since maternal plasma is a mixture of fragments from the mother and fetus, we separate the two components *in silico* and compare the aforementioned biological signatures to assess the differences between maternal and fetal DNA.

## Methods

### Ethics statement

This study was performed on raw sequencing data published by Kitzman et al. in their 2012 work of non-invasively sequencing the fetal genome [49]. Please refer to this paper for the original ethics. This data can be retrieved at the dbGaP archive [51] under project accession number [dbGap:phs000500.v1.p1]. A National Human Genome Research Institute (NHGRI) Data Access Committee assessed and approved the project request submitted by the authors. All samples were anonymized and no further ethics approval was needed prior to the use of the data.

### Study design and datasets used

The main dataset (I1_M) we used consists of two samples of DNA from a pregnant female: cell-free DNA from plasma (I1_M_plasma) and cellular DNA from leucocytes (I1_M_cellular). A second pair of matched plasma and cellular samples from a separate pregnancy (G1_M_plasma, G1_M_cellular) published by Kitzman et al. was used to replicate the major findings of this study. Both subjects I1_M and G1_M carried male fetuses at gestation ages of 18.5 and 8.2 weeks, with fetal fractions estimated by the original study to be around ~13 and ~7 % respectively.

The details of the extraction, purification and preparation of sequencing libraries for the DNA are provided in Kitzman et al. [49]. It is of note that during library preparation only the cellular DNA samples underwent fragmentation by sonication and subsequent size selection in the range of approximately 250 - 450 bp inclusive of adapters. These two steps were unnecessary for the biologically fragmented cell-free DNA in the plasma and were consequently by-passed, giving the opportunity to investigate fragment length distribution properties of cell-free DNA. All four DNA libraries underwent paired-end sequencing on the HiSeq 2000 instrument to generate paired-end reads of length 101 bp.

### Data processing

The read sequences in FASTQ format were aligned to the human genome, build 37 (hg19) using Novoalign V2.08.03 [52] with per-base quality score recalibration enabled. The genome reference used for mapping, had known SNPs encoded as IUPAC ambiguous codes to minimize the different alleles generating mismatches, which is particularly advantageous with the increased heterogeneity stemming from the mixture of fetal and maternal DNA. Novoalign was chosen instead the BWA mapper used in the original study, as it is implements a more sensitive and accurate alignment algorithm that also allows for the usage of an ambiguous reference allowing the alignment of more reads with greater specificity.

Reads that mapped to multiple locations and read pairs that were designated as PCR duplicates were discarded from each dataset using a combination of SAMtools V0.1.18 [53] and Picard software [54]. Local realignment around indels was performed *via* the GATK software suite [55] as a final read-processing step.

Unless otherwise mentioned, all proceeding analyses were carried out on fragments separated by chromosomal origin (by autosomes and mitochondrial DNA). When paired-end information was needed, only paired-reads that were correctly oriented with insert sizes comparable with the expected variation in each DNA library were used for analysis.

Only reads with mapping quality greater than the Phred-scale value of 13 were used, which corresponds to less than 5 % probability that the read is wrongly mapped. Custom R [56] and python scripts were written for analyses and any specific R packages used are stated in the text.

### Analyzing genome-wide and inter-chromosomal fragment length distributions

The DNA fragment lengths were inferred from the paired reads to examine the exact lengths present in each dataset. We performed Fourier analysis on the cell-free DNA fragment length density in the range 50 - 450 bp using the fast Fourier transformation as implemented in the *spectrum* package in R. This technique was used to de-convolve any complex periodicities present in the distribution of fragment lengths into a combination of simple periodic waves. We then analyzed the power spectrum of single frequencies *via* a periodogram to determine important frequencies that could explain the oscillation pattern of the observed data.

In order to assess the multi-modality of the major peaks in the cell-free DNA fragment length distribution, we fitted a 3-component Gaussian mixture model to the inferred lengths in both a genome-wide and per chromosome basis. The maximum likelihood approach was used to estimate the model parameters *via* the expectation maximization algorithm. Initial estimates for the means, standard deviations and proportions of the three underlying distributions were determined from the observed data. Quantiles of the theoretical and empirical genome-wide fragment length distributions were plotted to assess the fit of the model. We then visually examined the mixing probabilities assigned to the three components between all chromosomes to investigate any inter-chromosomal imbalance in the three subgroups of fragment lengths in cell-free DNA.

### Analyzing intra-chromosomal fragment length distributions

Since multiple prior studies have reported over- and under-represented repetitive regions in cell-free DNA [24–27], we investigated differences in fragment lengths originating at these sub-chromosomal regions. Repetitive locations in the human genome are curated in the Repbase database [57] under a ‘class/family/type’ classification with ‘type’ being the most specific grouping. The RepeatMasker annotation that draws on Repbase (Repeat Library 20090604) categorizes repeats into 57 unique ‘class/family’ combinations and 1395 unique repeat ‘types’. We first separated cell-free DNA fragments into the ‘class/family’ categories depending on if the 5′ end of the fragment mapped to these regions on the hg19 genome reference. We retained 32 of the most abundant categories that had at least 10,000 sequenced fragments aligning to them with mapping quality > = 13 and visually compared the fragment length profiles. To carry out a more specific interrogation of these regions, we used pattern matching of the repeat ‘type’ names to collapse them into more general categories and compared their fragment length profiles. Fifty of these repeat categories were chosen, mainly based on their genome-wide abundance in order to have enough power for the fragment length analysis. Certain categories of low abundance were also included to have a fair representation of the different classes of repetitive elements as specified in Repbase.

### Summarizing the higher-order genomic enrichment of cell-free DNA

We carried out strand cross-correlation analysis [58] to capture recurrent events of read coverage along the genome in the plasma and cellular datasets; with the hypothesis that the non-random nature of cell-free DNA cleavage would lead to clusters of reads that would not be present in cellular DNA sequencing data. To this effect, one read from each read pair was randomly sampled to simulate single-end read data. The Pearson correlation between the per-base coverage of the forward and reverse strands was calculated, each time shifting the reverse strand by increments of 1 bp (beginning from a lag of 20 up to 5000 bp). The cross-correlation value per strand-shift was plotted and compared between each sample.

Annotation from the ENCODE consortium was used to investigate the relationship between the read density signal in plasma data and chromatin higher-order structure. The randomly fragmented cellular data was used as a control. Nucleosome occupancy annotation for different cell-types was generated by the ENCODE project with the use of MNase-seq data where micrococcal nuclease (MNase) was used to isolate the DNA fragments bound to histones and subjected to high-throughput sequencing. Subsequent alignment of these fragments generated a nucleosome map. Open-chromatin regions were mapped using FAIRE-seq, where DNA was randomly fragmented using sonication. Subsequent formaldehyde assisted cross-linking of histones separated out the nucleosome-bound DNA from the nucleosome-depleted chromatin [59]. The latter fraction of fragments were sequenced and mapped to the human genome. These data were downloaded as uniformly processed signal files with normalized scores of nucleosome-occupancy (MNase-seq) or depletion (FAIRE-seq) for each base. Further information for these annotation tracks can be found at the ENCODE data portal [60].

All annotation was selected for the Gm12878 lymphoblast cell-type, which is one of three highly curated Tier 1 cell-lines provided by the ENCODE project and most closely matched the cell-free DNA, which is predominately of hematopoietic origin [61]. More information on this cell type can be found from the ENCODE project website [62].

The sequencing data from the I1_M and G1_M samples were converted into signal tracks using the Wiggler software [63], which is the official tool used by the ENCODE project to create the genome-wide MNase-seq and FAIRE-seq tracks used in the analysis. In brief, the software counts the read coverage per base along the genome and calculates a signal value by smoothing these counts using a Tukey kernel. The strand specific signal values are summed at each position and the final signal is corrected according to the mappability of the genomic regions.

The signals from ENCODE and the empirical tracks generated from the cell-free DNA data were binned into non-overlapping windows of 1 Kb by assigning the average signal value of all positions in the window. The window size is informed by the cross correlation analysis result that gives insight into the degree of distribution and consistency of read clustering patterns along the genome. Pearson correlation was calculated for each pair of binned datasets (2 ENCODE tracks, 4 plasma/cellular samples, thus a total of 8 comparisons) and the resulting correlation matrix was visualized using the *corrplot* package in R. To avoid outlier values in the sequencing signal tracks from unnecessarily affecting the correlation, we trimmed 10 % of the largest absolute residual values from a linear model fit to this data during each pair-wise analysis.

### Analysis of nucleotide signature at fragmentation sites

The proportions of each nucleotide (A, T, C, G) in an interval surrounding fragment start sites (+/- 25 bp) were calculated to examine if the breaks in cell-free DNA are random and independent of the underlying sequence. These position-specific proportions were then normalized by the genome-wide expected values for each type of mononucleotide to assess the relative frequencies. Fragments were then stratified according to their length into two intervals of [100, 140] bp and [200, 250] bp before repeating the previous analysis to investigate any size specific nucleotide signatures. The intervals were chosen with the prior knowledge that these fragment lengths would have a high likelihood of representing cleavage within nucleosomes (interval 1) and cleavage within linker DNA regions (interval 2).

We then used the *de novo* motif discovery software DREME [64] to mine the cell-free DNA data for motifs related to nuclease cleavage. Ten million fragments were randomly sampled from each plasma dataset and the 2 bp sequence on either side of the 5′ fragment ends were input into the software as the test sequences, each 5 bp in total length. The decision to limit the search to short DNA motifs was motivated by our previous work [50] which investigated the most influential positions around the cleavage sites in cell-free DNA. Similarly, 10 million sequences of identical length from the matched cellular data were input as the negative sequences, which were unlikely to contain motifs of interest. The program uses a Fisher’s exact test to determine the significance of each motif found in the test set as compared with its representation in the negative set.

### Investigating fetal-specific cell-free DNA characteristics

As previously described [65], SAMtools software was used to infer the genotypes at ~3 million HapMap Phase II SNPs for the matched plasma and cellular data separately. We identified all SNPs in the cellular samples where the mother was homozygous (AA) and selected the informative subset of these SNPs in the matched plasma sample, which exhibited the alternate allele originating from the fetus (i.e. fetus was heterozygous (AB) for the genotype). The paired-reads corresponding to the DNA fragments carrying the fetus specific alleles were separated out from the plasma data. The remaining fragments carrying the shared allele were used as maternal DNA by reason of the low fetal fraction in these samples. While the original study by Kitzman et al. did contain paternal sequencing data, there were concerns of poor quality noted in the original study since it was derived from saliva. Thus, we chose not to utilize the paternal information to further filter fragments carrying the shared allele.

Analysis of the fragment lengths and mononucleotide signature at the cleavage sites was carried out to document the differences between the fetal and maternal fragments. Separating the two components using alleles leads to fragment locations being restricted to those in the vicinity of informative SNPs and a relatively small number of eligible fragments for the fetus. Therefore, certain genome-wide investigations were ruled out and the analysis scope was limited to the two avenues stated previously.

## Results

### Sequencing coverage statistics

All subsequent results are based on matched cellular and cell-free plasma DNA from two pregnancies (I1_M and G1_M) that have undergone 101 bp paired-end sequencing on the Illumina HiSeq platform. Table 1 provides the clinical details and naming conventions for the relevant datasets as provided by Kitzman et al. [49].

**Table 1:** Details of datasets used in study

Sample name	Type of DNA	Fetal karyotype	Gestational age	Fetal DNA fraction
I1_M_plasma	Cell-free	46, XY	18.5 weeks	~13 %
G1_M_plasma	Cell-free	46, XY	8.2 weeks	~7 %
I1_M_cellular	Cellular	-	-	-
G1_M_cellular	Cellular	-	-	-

Table 2 specifies the number of sequencing reads at each step of the data-processing pipeline and provides the whole-genome coverage for each dataset. Eight percent of sequencing reads are unable to be aligned in plasma as opposed to ~3 % in the cellular datasets. Plasma DNA also shows a higher number of PCR duplicates compared to cellular (3 % *vs*. 0.5 %), which is expected, given the extra PCR cycles in the library protocol when using a low starting volume of DNA. However, similar proportions of multi-mapping reads (3-4 %) were obtained for all datasets regardless of the origin of the DNA. After read filtration, I1_M has very high genome-wide counts for cell-free plasma DNA with a mean coverage of 74 X (2.2 billion reads) and 32 X for the cellular component. G1_M gives 52 X mean coverage (1.6 billion reads) for plasma and 27 X for cellular data.

**Table 2:** Alignment and processing statistics for sequencing read data

	I1_M_plasma		G1_M_plasma	
	Number of reads	Proportion of total	Number of reads	Proportion of total
Total	2663662496	1	1920061404	1
Aligned	2462191486	0.92	1758285925	0.92
Uniquely aligned	2366811398	0.89	1686208471	0.88
Non-duplicates	2312044964	0.87	1641590824	0.85
MAPQ > = 13	2225473345	0.84	1585773977	0.83
Proper pairs	1112724850	-	792884037	-
Whole-genome coverage	74.2 X		52.6 X	
	I1_M_cellular		G1_M_cellular	
	Number of reads	Proportion of total	Number of reads	Proportion of total
Total	1091176962	1	903802456	1
Aligned	1056822490	0.97	888679846	0.98
Uniquely aligned	1023380388	0.94	860508538	0.95
Non-duplicates	1019233466	0.93	858226379	0.95
MAPQ > = 13	983181449	0.90	838372718	0.93
Proper pairs	491581650	-	419209864	-
Whole-genome coverage	32.6 X		27.4 X	

**Fig. 1:**
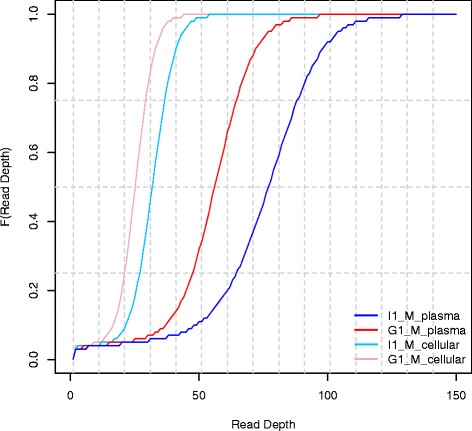
Empirical cumulative distribution functions of per-base read coverage for matched cell-free DNA and cellular samples. The two cfDNA datasets are named I1_M_plasma and G1_M_plasma while the cellular DNA from the matched subjects are named I1_M_cellular and G1_M_cellular

Figure 1 presents the empirical cumulative distribution function of the total read depth at genomic positions for the 4 datasets. There is greater variation in per-base coverage between the two plasma datasets than their matched cellular counter parts. For example, 75 % of the bases in I1_M_plasma is covered by 87 reads or less compared to 63 in G1_M_plasma, while for I1_M_cellular and G1_M_cellular the values are around 35 and 28 respectively.

### Genome-wide and inter-chromosomal fragment length distributions

**Fig. 2:**
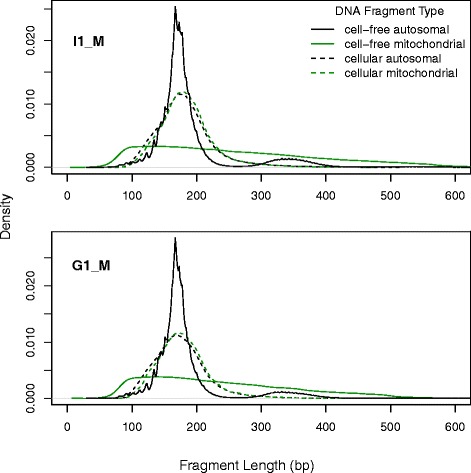
Size distributions of cell-free DNA contrasted with cellular DNA for two subjects (I1_M and G1_M). Fragments are divided into autosomal and mitochondrial classes and fragment sizes are calculated using the paired-positioning of sequencing reads

The fragment length density for cellular DNA is uni-modal, with a narrow range of sizes (first quartile = 153 bp, median = 177 bp, third quartile = 202 bp). There is no discernible difference between the cellular autosomal and mitochondrial profiles (Fig. 2). The cellular data mode at ~182 bp is due to the random nature of DNA cleavage and subsequent size selection that fragments undergo at library preparation.

Cell-free autosomal DNA in contrast, presents two clear modes at approximately 167 bp and 340 bp with a much wider mode around 510 bp. These three peaks appear to correspond to the lengths of DNA associated with a mono-, di- and tri-nucleosome structure respectively.

While the first cell-free DNA autosomal mode peaks at ~167 bp, it also shows minor peaks at roughly 145, 134, 123, 113, 102, 92 and 82 bp (Additional file 1). Three more signals are visible at 151, 173 and 177 bp. The fragment lengths appear to exhibit an approximate periodicity of 10 bases below 145 bp. This periodicity decreases in longer fragments and in the second mode the periodicity decreases to ~ 5 bp although it is not as strong or consistent as in the first major peak. This is possibly due to the substantially low number of observations in longer fragment lengths.

The spectral analysis of the fragment length distribution shown in Additional file 2 confirms these observations. The first two dominant frequencies occur at 0.00556 and 0.092 and correspond to periodicities of 180 and 10.9 bp on the reciprocal scale. Several signals smaller than 10 bp also appear in the Fourier analysis, which appears to reflect the high frequency pattern visible in fragment lengths longer than 145 bp.

It should also be noted that in I1_M and G1_M samples the mode in the matched cellular DNA corresponds to the first mode of cell-free DNA autosomal fragments by chance as it is only due to the specific sizes selected in the library preparation.

In contrast to the autosomal cell-free DNA, fragments mapping to mitochondrial DNA lack the 3-mode signature and exhibited a wider range of sizes. This observation may relate to the absence of higher-order packaging in the circular mitochondrial DNA, leaving it more exposed to enzymatic cleavage. This further adds evidence for the hypothesis that it is the nucleosome packaging and the approximately 10 bp 360° turn of the double helix that are key determinants for the fragmentation of autosomal cell free DNA.

As Additional file 3 shows, a 3-component Gaussian mixture provides an adequate fit for the autosomal fragment lengths when only modeling the tri-modal nucleosomal signal. The estimates for the genome-wide proportions of the mono-, di- and tri-nucleosomal distributions are 0.88, 0.11 and 0.01 for I1_M_plasma and 0.90, 0.09 and 0.01 for G1_M_plasma. The corresponding means of the three component distributions for I1_M are 169, 341 and 508 bp with the distributions exhibiting standard deviations of 24, 35 and 43 bp. G1_M has estimated means of 167, 337 and 492 bp with standard deviations of 23, 36 and 42 bp for the 3 components respectively. Full details of the genome-wide and per-chromosome maximum likelihood estimates of the distribution parameters are presented in Additional file 4.

**Fig. 3:**
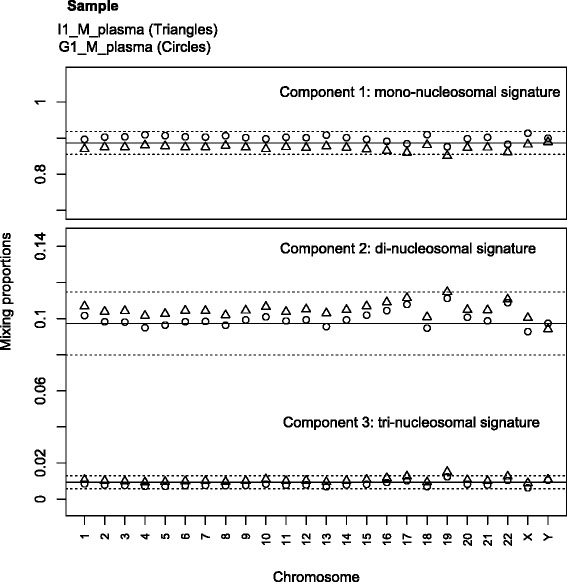
Estimated proportions from the 3-component Gaussian mixture model of the cell-free fragment lengths separated by chromosome. For both samples I1_M_plasma and G1_M_plasma, these estimates approximate the proportion of mono-, di- and tri-nucleosome lengths in each chromosome. All other mixture model parameters are reported in Additional file 4. The solid lines depict the average value in each component while the dashed lines demarcate +/- 3 standard deviations from the mean

An inter-chromosomal comparison shows that the mixing proportions of the model components reveal no gross imbalances in the 3 fragment length groups between the chromosomes (Fig. 3), with the largest variation occurring in the proportion for the second component.

### Intra-chromosomal fragment length distributions

Cell-free DNA has previously been shown to exhibit higher proportions of repeats such as SINEs and micro-satellites and decreased amounts of LINE elements [24, 25]. It is interesting to note that LINE elements are mainly located in condensed heterochromatin and Alu repeats localize to more open euchromatin regions. Whether DNA fragments from these repeat regions are released in these unbalanced proportions or specific cell-free DNA clearing mechanisms maintain the under/over-representation is currently not well understood. We investigated fragment lengths originating at different repeats to gain an insight into this imbalance hypothesizing that any preferential enzymatic clearing mechanisms could also affect the size of the DNA molecules containing specific repeats.

**Fig. 4:**
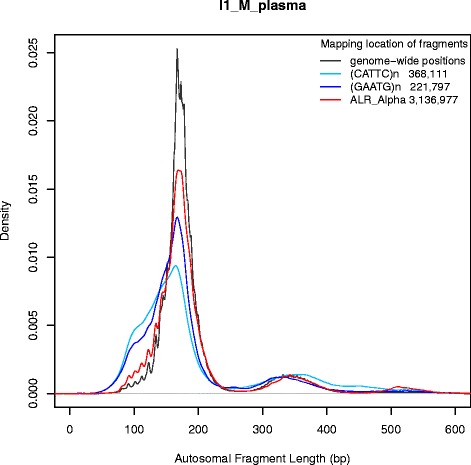
Autosomal fragment lengths originating at regions annotated for alpha repeat elements and two micro-satellite types. The number of fragments used to calculate the size distribution is depicted in the legend beside each repeat category. The repeat specific profiles are superimposed over the genome-wide profile in sample I1_M_plasma for comparison purposes

We make use of the Repbase database and RepeatMasker annotation for this analysis [57]. When comparing 32 broad categories of abundant repeats as per the class/family classification in RepeatMasker, there appears to be no difference in the fragment sizes with the density curves overlaying each other closely (Additional file 5). Narrowing the scope to 50 more specific repeat types within the broader classifications also shows no gross imbalances in fragment lengths except in three categories (Fig. 4). Details for the 50 repeat types analyzed are provided in Additional file 6.

Figure 4 compares the lengths of fragments mapping across the genome to those originating from specific regions containing micro-satellites annotated as (CATTC) n and (GAATG) n as well as regions of alpha repeat elements. More than 200,000 fragments are used to create the density curves for each micro-satellite and more than 3 million fragments are used for the more abundant alpha repeats. The two groups of microsatellites clearly show smaller fragment sizes when compared to the genome-wide profile. It is of note that the GAATG motif is the reverse-complement of CATTC generating the hypothesis that both strands are affected by a similar cleavage process to produce this divergence in fragment lengths. Alpha repeats in contrast appear to show more enrichment than expected in the third mode that corresponds to the tri-nucleosome lengths suggesting that they are protected from nucleases. None of the other 47 types showed a notable deviation in their fragment length profiles (results not shown).

### Higher-order genomic enrichment of cell-free DNA

**Fig. 5:**
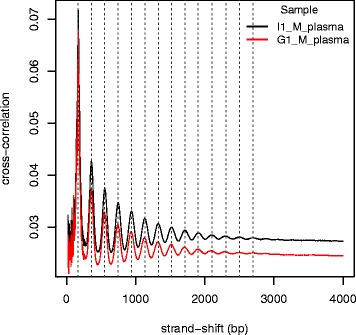
Strand cross-correlation analysis for cell-free DNA. The 3′ strand is shifted with respect to the forward strand in increments of 1 bp and the Pearson’s correlation between the per-position read counts for each strand is calculated to generate this cross-correlation plot

Cell-free DNA autosomal fragment lengths show clear signs of nucleosome related cleavage (Fig. 2). We further investigate this non-random cleavage by examining sequencing-read coverage patterns along the genome using strand-cross correlation analysis [58]. The cross-correlation plot for cell-free DNA (Fig. 5) shows a strong periodicity that gradually decreases in amplitude but nevertheless extending over a region up to 3000 bp. Overall, the strength of the correlation is a function of the depth of sequencing as evidenced by the decrease in values for G1_M (52 X) compared to I1_M (74 X). The different signals contributing to this pattern is described below.

The best overlay (highest correlation) between the reads on the forward and reverse strands, when they are shifted with respect to each other, occurs at 167 bp. This corroborates the dominant fragment length in Fig. 2. The corresponding cross-correlation plot for the cellular data only shows one signal and it is at 177 bp, which corresponds to the median cellular fragment length (Additional file 7).

In cell-free DNA, high correlation between the read counts of the two strands recur at multiple distances of ~190 bp from each other. These recurring peaks suggest that cell-free DNA reads occur in equidistant clusters. Therefore, the cross-correlation analysis suggests that paired reads across the two strands are separated by distances equivalent to the fragment lengths present (the most prevalent being 167 bp) and the reads on the same strand are separated by ~190 bp. This pattern can be observed to extend up to 3 Kb. This regularity of coverage enrichment is not present in the randomly fragmented cellular data as there is no periodicity in the correlation signal (Additional file 7).

**Fig. 6:**
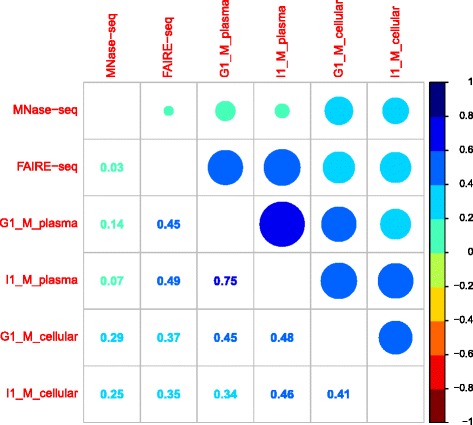
Pearson’s correlation of cell-free and cellular DNA read coverage signal with open/closed chromatin enrichment annotation. Pairwise Pearson’s correlation is calculated between fragment start site signal tracks from cell-free and cellular DNA sequencing data along with open chromatin (FAIRE-seq) and nucleosomal position (MNase-seq) signal annotation from ENCODE. The figure provides the pictorial representation of the resulting correlation matrix

To investigate the relationship of these read clusters with higher-order chromatin organization we downloaded annotation tracks that give signal strength for stable nucleosome cores (MNase-seq) and open chromatin regions (FAIRE-seq) in the lymphoblast cell-line (Gm12878) available through the ENCODE project [59, 63]. For each plasma sequencing dataset, we converted the read coverage at each position along the chromosomes into a window-based signal, utilizing the software used to generate the ENCODE signal tracks. The cellular samples underwent the same process to act as controls. Subsequently, pairwise Pearson correlations were calculated for the signal values between the 6 tracks (I1_M_plasma, G1_M_plasma, I1_M_cellular, G1_M_cellular, MNase-seq, FAIRE-seq). Fig. 6 provides the pictorial representation of the resulting correlation matrix.

The results show that cell-free DNA fragment-end positions are moderately correlated with the open chromatin regions in the annotation (Pearson’s correlations of 0.45, 0.49) while these cleavage positions have little to no correlation with the nucleosomal core positions (0.14, 0.07). In contrast, cellular DNA with its random fragment positions show low correlations in the range of 0.25 – 0.37 with both MNase-seq and FAIRE-seq signals. There also appears to be very little correlation between nucleosome position and open-chromatin signal tracks although we would expect a negative correlation. The non-random nature of cleavage in cell-free DNA is highlighted by a high-correlation in the coverage signal between the two plasma datasets (0.75), while the cellular DNA samples only shows a moderate correlation with each other (0. 41).

This analysis was conducted to gain an overall understanding of the cleavage patterns of cell-free DNA fragments along the genome. The experimentally derived annotations of the genome that describe open and closed chromatin states that were used in this analysis are noisy signals. Despite this, the results support the hypothesis that the structure in plasma sequencing data correlates with known biological signals. This was evidenced by both cross-correlation analysis using read-depth measures and genomic co-location of fragment ends with related ENCODE annotation.

### Nucleotide signature at fragmentation sites

**Fig. 7:**
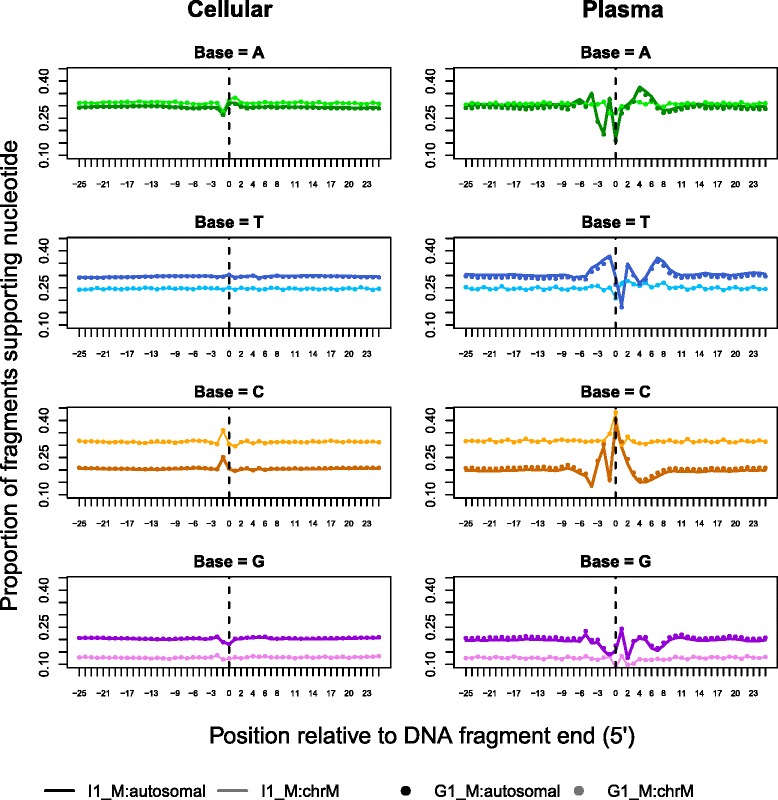
Mononucleotide frequencies for the region of 51 bp (+/−25 bp) around fragment start sites. The y-axis denotes the proportion of each nucleotide at fixed positions relative to the 5′ end of the DNA fragment and the vertical line at 0 denotes the fragment start. Sample I1_M is denoted with lines while circles represent the G1_M values. For both cellular and cell-free data in the two samples, fragments are divided into autosomal and mitochondrial classes displayed in dark and light colors for each base respectively

In this analysis, we examined the base proportions around the read starts in the I1_M and G1_M sequencing data. Cellular DNA does not show a dependence on specific nucleotides for the region surrounding the fragment break (position 0 in Fig. 7) except for a small preference for Cytosine at a position in the reference genomic sequence adjoined to the 5′-end of the read (position -1). This appears to be a technical bias related to the shearing process involved in the Covaris instrument used to fragment the cellular DNA [66]. The base-preference per position is very similar between the autosomal and mitochondrial components of the cellular data except for the difference in average proportion of the bases due to one of the strands in mitochondrial DNA being Cytosine-rich (referred to as the heavy strand).

In contrast to cellular DNA, the nucleotide proportions for cell-free DNA autosomal fragments show a clear position specific pattern with Cytosine taking prominence at positions 0 (cleavage site), 1 and -2. The pattern extends up to ~10 bp on either side of the site as seen in Additional file 8, which gives the relative frequencies of the nucleotides at each position. We also note that the nucleotide signatures in G1_M and I1_M are very similar to the patterns observed in 29 low-coverage (>0.5 X) datasets that we have published previously [50]. The low-coverage datasets are generated from independent samples using different sequencing platforms and different versions of library-prep kits. Since this analysis reproduced the nucleotide pattern, it appears to give further evidence that this signature is not a technical artifact of the downloaded data and strongly indicates a biological origin.

Compared with the result in autosomal fragments, cell-free mitochondrial DNA shows a noticeable lack of perturbation in base proportions except at the cleavage site where it shows a small preference for Cytosine. The differences between the autosomal and mitochondrial profiles appear to connect back to the fragment size differences seen in Fig. 2 and the higher-order structural differences between the two categories of DNA.

We do not observe a notable difference in position specific nucleotide preference when separating the fragments by size (Additional file 9). However, we do observe that fragments we inferred to be cleaved within the nucleosome subunit (lengths 100-140 bp) has higher proportions of G and C bases than those originating from cleavage at the linker DNA (lengths 200-250 bp) evidenced by the inversion of the marginal profiles between the two fragment classes. This observation is supported by previous work, which document that nucleosomal regions are generally GC-rich while linker regions are GC-poor [67, 68].

Moving on from the marginal nucleotide profiles, we investigate the joint distribution of nucleotides at the cleavage site by looking for short sequence motifs with differential enrichment between cell-free and cellular DNA. Additional file 10 presents the top result for both I1_M and G1_M. This motif corroborates the marginal profiles in Fig. 7 in that nucleotide C takes prominence in both the 0^th^ and 1^st^ position at the cleavage site. Bases C, G and T are preferred over A in position -2 and C, T, A nucleotides are preferred over G at position 2. There is little support for a specific base at the position immediately before the fragment start (-1). This motif was the top result in both datasets with ~1.5 million cell-free DNA sequences out of 10 million supporting the full 5 bp motif compared to ~0.5 million in cellular DNA.

### Comparison of maternal and fetal fragments

Sample I1_M has 26,162 SNPs that were genotyped with high confidence as homozygous in the mother (I1_M_cellular) and heterozygous in the cell-free DNA mixture (I1_M_plasma). Close to 224,000 fragments carry the fetal-specific allele at these informative SNPs and 1,749,269 fragments carrying the shared allele were classified as maternal for analysis purposes. Sample G1_M only contained 7497 informative SNPs due to its lower coverage and we separated out 351,648 maternal and 22,843 fetal fragments.

**Fig. 8:**
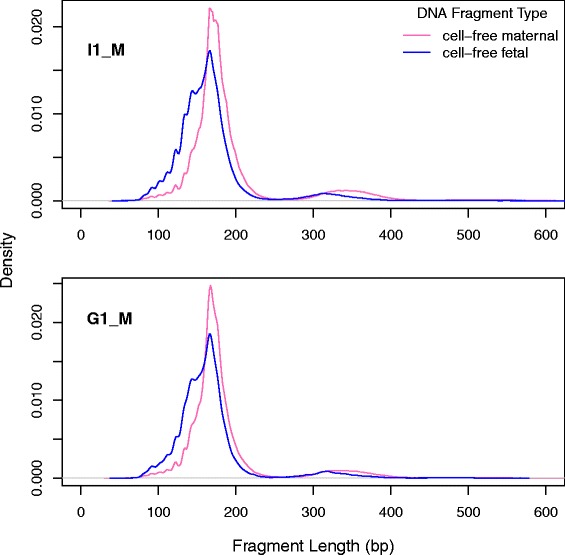
Size distributions of maternal cell-free DNA contrasted with fetal DNA for two subjects (I1_M and G1_M). Fragments are classed into the two components using allelic information at informative SNPs. Fragment sizes are calculated using the paired-positioning of sequencing reads

When comparing the fragment length profiles between these two components (Fig. 8) we see that the fetal distribution is shifted toward the shorter end, compared with the maternal distribution. Table 3 provides summary statistics for the two classes of fragments and shows that the median maternal fragment length in I1_M and G1_M is 174 and 171 bp respectively while the median for the fetal component is ~160 bp. We see that the fetal-specific signal is depleted for the di-nucleosomal peak (third quartile) and there are more fetal sequences with lengths shorter than that of a mono-nucleosome (first quartile for maternal fragment sizes in I1_M and G1_M is 162 and 159 bp respectively while the fetal values are calculated to be 141 and 142 bp).

**Table 3:** Summary statistics for maternal and fetal fragment lengths

Statistic	I1_M		G1_M	
	Maternal	Fetal	Maternal	Fetal
Q1	162.0	141.0	159.0	142.0
Median	174.0	160.0	171.0	161.0
Mean	191.5	166.9	185.0	166.6
Q3	191.0	176.0	186.0	175.0
Standard dev.	66.3	51.0	59.9	50.4

**Fig. 9:**
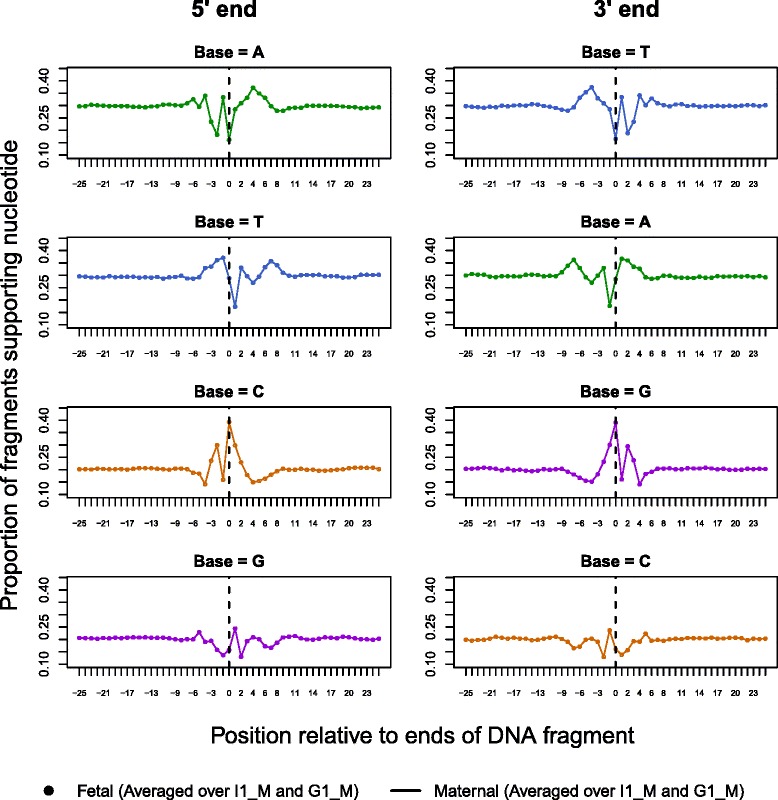
Comparison of the nucleotide signature at fragmentation sites for fetal and maternal fragments. This plot illustrates the mononucleotide frequencies for the region of 51 bp (+/−25 bp) around fragment starts and ends. The y-axis denotes the proportion of each nucleotide at fixed positions relative to the 5′ and 3′ ends of the DNA fragment and the vertical line at 0 denotes the strand specific fragment end. Maternal proportions per position are connected with lines while circles represent the fetal values. For both components, the proportions have been averaged over I1_M and G1_M. The close overlay of the fetal proportions and maternal values show that the variability between them is nearly negligible

Interestingly, even though there is a marked difference in size, the position specific nucleotide pattern is very similar between maternal and fetal fragments (Fig. 9). There is also no evidence for a strand specific fragmentation signature since the 3′ ends of the fragments show the reverse complement of the 5′ pattern.

## Discussion

In this study we interrogate multiple sequence signals that appear due to the non-random nature of cell-free DNA fragmentation using high coverage sequencing of maternal plasma.

By first examining the fragment lengths present in cell-free DNA and then investigating the position of fragments along the genome, we corroborate the leading hypothesis in literature [8, 28] that fragmentation is primarily between nucleosomes with subsequent intra-nucleosomal cleavage along the DNA helical turn. The lengths corresponding to one nucleosomal subunit appear to be the most prevalent and conserved size with di- and tri-nucleosomal lengths showing much lower proportions. We examine the periodicity in cell-free DNA fragment length distribution using Fourier analysis and confirm the ~180 and ~10 bp periodicity due to the two levels of cleavage which others have only assessed visually. In addition, because of the high coverage data, we are able to show that longer fragments do not exhibit this stable 10 bp pattern and certain lengths show a decreased periodicity of ~5 bp. Only around 10 % of the fragments are shorter than 145 bp, which is the range that exhibits the 10 bp periodicity most clearly. This length (~145 bp) is related to DNA wrapped around the nucleosome (excluding the DNA connected to the peripheral histone H1 which adds ~20 bp). The lack of fragments in this range could be due to the rapid enzymatic activity once the DNA wrapped around the histones are exposed to cleavage. Hence, cell-free DNA associated with one full nucleosomal subunit (~167 bp) appears to be preferentially protected from further enzymatic cleavage and creates a stabilizing structure in circulation evidenced by its prevalence in the fragment length distribution. We also show that these sequence signatures are completely missing from mitochondrial DNA that lacks the higher-order packaging which nuclear DNA undergoes, lending more evidence to the hypothesis of nucleosome-related cleavage.

While there is no major difference in the fragment length distributions between chromosomes and in different repeat categories within chromosomes, our analysis illuminated a few exceptions. Fragments containing a repeating motif of CATTC and reverse complement GAATG show higher than average rate of cleavage. Previously, these repeats had been shown to be over-represented in the cell-free DNA from apoptotic human umbilical-vein endothelial cells [25]. Since the other simple repeats analyzed did not show this difference in fragment lengths, our observations would indicate that this motif is specifically involved in the biological processes that produce cell-free DNA. Investigating this rationale further is beyond the scope of this study although this observation maybe useful in cancer research which uses cell-free DNA micro-satellite instability as a biomarker for presence of tumor DNA. We also observed that fragments with sizes in the order of tri-nucleosomal DNA were enriched for alpha repeats. It can be hypothesized that since alpha repeats generally occur in heterochromatin regions, the longer fragment lengths are due to the regions being generally inaccessible by enzymes due to the dense packaging.

Utilizing cross-correlation analysis, we showed that cell-free DNA exhibited highly regular spacing of sequence read-counts, where fragment end coverage alternates between high and low in neighboring regions corresponding to the ‘beads-on-a-string’ nature of consecutive nucleosomes in stretches up to 3 Kbp. This gives cell-free DNA sequencing data remarkable structure in terms of read coverage across the genome.

Utilizing ENCODE annotation we showed that the cell-free DNA fragment starts and ends are more correlated with open chromatin regions than nucleosomal cores, corroborating the previous observations that the DNA is cleaved at nucleosome linker regions. This analysis is an approximate examination of the non-random cleavage patterns of cell-free DNA fragments along the genome. While ENCODE provides the most curated annotation, it is highly likely that it only represents a fraction of the chromatin elements in the genome as it requires concordance between data from different replicates, different laboratories of origin etc. Furthermore, the lymphoblast cell-type used may not be ideal for cell-free DNA in blood, which is almost certainly a mixture of fragments from varying tissue origin [69]. These inconsistencies, general noisiness in sequencing data when averaging across the genome and read coverage imbalances between samples needs to be taken into account and could explain the only modest difference between plasma cell-free DNA and cellular data for open chromatin.

However, these two analyses show that coverage in cell-free DNA sequencing data does not vary simply due to technical biases such as GC-content and read mappability along the genome but also due to biological factors. This is important in copy-number variation analysis where CNVs in certain regions would be harder to detect simply due to lack of cell-free DNA fragments originating from these regions despite the overall depth of sequencing. Benjamini and Speed [70] showed that incorporation of such fragment features and coverage patterns was possible and improved CNV detection for cellular DNA data. Our previous work [50] also showed that even using only some of the biological signals detected in this work already lead to a substantial improvement in trisomy 21 detection. Therefore, it is likely to be beneficial for other bioinformatics algorithms to take this fine-scale structure in the sequencing data into account to avoid this biological bias in coverage.

In a novel result, we showed that cell-free DNA cleavage is sequence dependent where mononucleotide frequencies show a consistent position specific pattern in the region spanning up to 10 positions on either side of the DNA cleavage site. This marginal sequence motif is similar in nucleosomal core and linker regions but is absent in nucleosome-free mitochondrial DNA. The pattern we see at the cleavage site could be the final result from a complex mixture of cell-free DNA in circulation due to different factors i.e. different proportions of apoptotic/necrotic input, endo- and exo- nuclease activity and different tissue origin. However, this specificity of nucleotides at positions around the breakpoint has implications for sequence motifs and is a potential source of variability that can be used to compare between different diseased states in cell-free DNA biomarker analysis.

Although we analyzed the above sequence signatures using the cell-free DNA mixture in maternal plasma as a whole, we also separated the two components belonging to the mother and fetus. Our work corroborated observations by others [8, 28] that showed that fetal DNA tends to be shorter than maternal DNA. However, we have now shown that both fetal and maternal cell-free DNA components are affected by comparable enzymatic or biological processes due to the similarity in the nucleotide signature at the fragment ends. Since cell-free DNA fragmentation mechanisms are not fully understood we can only speculate that perhaps shorter fragments are preferentially released into circulation from fetal cells or that fetal DNA is not as well packaged as maternal DNA leaving it more exposed towards enzymes in blood and thus producing shorter fragment lengths.

## Conclusions

In recent years, high-throughput sequencing of cell-free DNA has revolutionized prenatal testing by providing a more accurate non-invasive screening method for fetal aneuploidy. Cell-free DNA also shows great promise as a source of data to detect early signs of cancer, interrogate the genetic landscape of tumours and track the evolution of associated mutations after treatment. However, clinical research has been impeded by the lack of knowledge on the biology of this extracellular DNA and the low signal to noise ratio in the procured data.

**Fig. 10:**
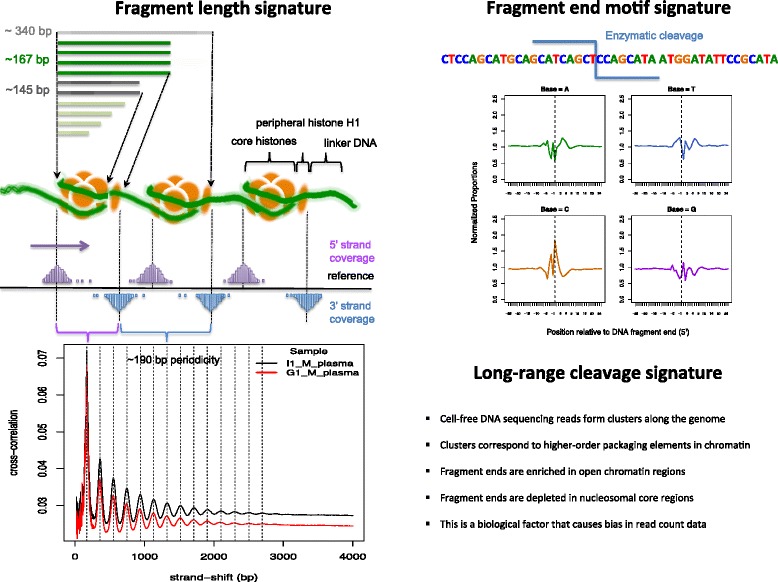
Summary of the main sequence signatures and underlying biological signals documented by the study

Here we show that there is considerable biological background signal (Fig. 10) in cell-free DNA sequencing data that could be harnessed to improve existing bioinformatics analysis as well as providing reproducible biological variation, which, when taken into account, should improve detection methods in particular for copy number variation.

The landscape of cell-free DNA in circulation would differ in various pathological conditions due to differences in apoptotic and necrotic contributions to the total pool of cell-free DNA, the behavior of the immune system or epigenetic changes resulting in alterations of chromatin structure [71]. In this work we have discovered potential sources of variability in cell-free DNA data. These, along with the descriptive measures we implement can be used to characterize the changes in cell-free DNA occurring in the aforementioned situations. Our observations using cell-free DNA in plasma can also be used as a base line in biomarker studies to compare and contrast between extracellular DNA from different sources such as urine, synovial and cerebrospinal fluids.

## Additional files

Additional file 1: Figure S1. Size distributions of autosomal cell-free DNA from two subjects (I1_M and G1_M). The plots show the ~ 10 bp periodicity in fragment lengths smaller than 145 bp and the approximate 5 bp periodicity in fragment sizes between 290−390 bp. Additional file 2: Figure S2. Smoothed periodogram of the time-series fit to the cell-free autosomal fragment lengths of samples I1_M and G1_M. Additional file 3: Figure S3. Goodness of fit of the 3-component Gaussian mixture model fitted for the genome-wide autosomal fragment lengths in cell-free DNA data. While the distribution function of the mixture is the sum of weighted Gaussian probabilities, its inverse is computed numerically. Additional file 4: Table S1. Estimated parameter values from the 3-component Gaussian mixture model. The model is fitted to the autosomal fragment lengths calculated in the genome-wide context and in each chromosome separately. Additional file 5: Figure S4. The density profiles of autosomal fragment lengths originating at 32 different repeat categories in sample I1_M_plasma. The number of fragments used to calculate the size distribution is depicted in the legend beside each repeat category. Additional file 6: Table S2. Specific repeat types examined for difference in fragment length densities. The information has been retrieved using Repeat Library 20090604 in the RepeatMasker annotation. Additional file 7: Figure S5. Strand cross-correlation analysis for cellular DNA. The 3′ strand is shifted with respect to the forward strand in increments of 1 bp and the Pearson’s correlation between the per-position read counts for each strand is calculated to generate this cross-correlation plot. Additional file 8: Table S3 Normalized mononucleotide frequencies for the region of 51 bp (+/−25 bp) around autosomal fragment start sites for cell-free DNA. Additional file 9: Figure S6. Mononucleotide frequencies for the region of 51 bp (+/−25 bp) around fragment start sites, separated by fragment length. The y-axis denotes the proportion of each nucleotide at fixed positions relative to the 5′ end of the autosomal DNA fragment and the vertical line at 0 denotes the fragment start. Values are averaged between I1_M and G1_M samples. Fragments are divided into two classes according to their length displayed as solid and dashed lines respectively. Additional file 10: Figure S7. Top result from the discriminatory sequence motif analysis using DREME software.

## Abbreviations

ENCODE: The Encyclopedia of DNA Elements
FAIRE: Formaldehyde-Assisted Isolation of Regulatory Elements
LINE: Long interspaced nuclear element
MNase: Micrococal nuclease
NGS: Next-Generation Sequencing
SINE: Short interspaced nuclear element

## References

[1] Mandel P, Metais P (1948). Les acides nucléiques du plasma sanguin chez l’homme. CR Acad Sci Paris.

[2] Jahr S, Hentze H, Englisch S, Hardt D, Fackelmayer FO, Hesch RD (2001). DNA fragments in the blood plasma of cancer patients: quantitations and evidence for their origin from apoptotic and necrotic cells. Cancer Res.

[3] Li Y, Zimmermann B, Rusterholz C, Kang A, Holzgreve W, Hahn S (2004). Size separation of circulatory DNA in maternal plasma permits ready detection of fetal DNA polymorphisms. Clin Chem.

[4] Stroun M, Lyautey J, Lederrey C, Olson-Sand A, Anker P (2001). About the possible origin and mechanism of circulating DNA: Apoptosis and active DNA release. Clin Chim Acta.

[5] van der Vaart M, Pretorius PJ (2007). The origin of circulating free DNA. Clin Chem.

[6] Choi J-J, Reich CF, Pisetsky DS (2005). The role of macrophages in the in vitro generation of extracellular DNA from apoptotic and necrotic cells. Immunology.

[7] Gahan PB, Anker P, Stroun M (2008). Metabolic DNA as the origin of spontaneously released DNA?. Ann N Y Acad Sci.

[8] Tsui NBY, Jiang P, Chow KCK, Su X, Leung TY, Sun H (2012). High resolution size analysis of fetal dna in the urine of pregnant women by paired-end massively parallel sequencing. PLoS One.

[9] Yu SCY, Lee SWY, Jiang P, Leung TY, Chan KCA, Chiu RWK (2013). High-resolution profiling of fetal DNA clearance from maternal plasma by massively parallel sequencing. Clin Chem.

[10] Liimatainen SP, Jylhävä J, Raitanen J, Peltola JT, Hurme MA (2013). The concentration of cell-free DNA in focal epilepsy. Epilepsy Res.

[11] Leon SA, Revach M, Ehrlich GE, Adler R, Petersen V, Shapiro B (1981). DNA in synovial fluid and the circulation of patients with arthritis. Arthritis Rheum.

[12] Sriram KB, Relan V, Clarke BE, Duhig EE, Windsor MN, Matar KS (2012). Pleural fluid cell-free DNA integrity index to identify cytologically negative malignant pleural effusions including mesotheliomas. BMC Cancer.

[13] Holdenrieder S, Nagel D, Schalhorn A, Heinemann V, Wilkowski R, von Pawel J (2008). Clinical relevance of circulating nucleosomes in cancer. Ann N Y Acad Sci.

[14] Holdenrieder S, Stieber P, Chan LYS, Geiger S, Kremer A, Nagel D (2005). Cell-free DNA in serum and plasma: comparison of ELISA and quantitative PCR. Clin Chem.

[15] Peters DL, Pretorius PJ (2011). Origin, translocation and destination of extracellular occurring DNA-a new paradigm in genetic behaviour. Clin Chim Acta.

[16] Zheng YWL, Chan KCA, Sun H, Jiang P, Su X, Chen EZ (2012). Nonhematopoietically derived DNA is shorter than hematopoietically derived DNA in plasma: a transplantation model. Clin Chem.

[17] De Vlaminck I, Valantine HA, Snyder TM, Strehl C, Cohen G, Luikart H (2014). Circulating cell-free DNA enables noninvasive diagnosis of heart transplant rejection. Sci Transl Med.

[18] Chan KCA, Jiang P, Zheng YWL, Liao GJW, Sun H, Wong J (2013). Cancer genome scanning in plasma: detection of tumor-associated copy number aberrations, single-nucleotide variants, and tumoral heterogeneity by massively parallel sequencing. Clin Chem.

[19] Dawson S-J, Tsui DWY, Murtaza M, Biggs H, Rueda OM, Chin S-F (2013). Analysis of circulating tumor DNA to monitor metastatic breast cancer. N Engl J Med.

[20] Korabecna M, Pazourkova E, Horinek A, Rocinova K, Tesar V (2013). Cell-free nucleic acids as biomarkers in dialyzed patients. JN.

[21] Zhong X-Y, von Mühlenen I, Li Y, Kang A, Gupta AK, Tyndall A (2007). Increased concentrations of antibody-bound circulatory cell-free DNA in rheumatoid arthritis. Clin Chem.

[22] Jensen TJ, Zwiefelhofer T, Tim RC, Dzakula Z, Kim SK, Mazloom AR (2013). High-throughput massively parallel sequencing for fetal aneuploidy detection from maternal plasma. PLoS OnE.

[23] Dan S, Wang W, Ren J, Li Y, Hu H, Xu Z (2012). Clinical application of massively parallel sequencing-based prenatal noninvasive fetal trisomy test for trisomies 21 and 18 in 11 105 pregnancies with mixed risk factors. Prenat Diagn.

[24] Beck J, Urnovitz HB, Riggert J, Clerici M, Schütz E (2009). Profile of the circulating DNA in apparently healthy individuals. Clin Chem.

[25] Morozkin ES, Loseva EM, Morozov IV, Kurilshikov AM, Bondar AA, Rykova EY (2012). A comparative study of cell-free apoptotic and genomic DNA using FISH and massive parallel sequencing. Expert Opin Biol Ther.

[26] Stroun M, Lyautey J, Lederrey C, Mulcahy HE, Anker P (2001). Alu repeat sequences are present in increased proportions compared to a unique gene in plasma/serum DNA: evidence for a preferential release from viable cells?. Ann N Y Acad Sci.

[27] van der Vaart M, Pretorius PJ (2008). A method for characterization of total circulating DNA. Ann N Y Acad Sci.

[28] Fan HC, Blumenfeld YJ, Chitkara U, Hudgins L, Quake SR (2010). Analysis of the size distributions of fetal and maternal cell-free DNA by paired-end sequencing. Clin Chem.

[29] Lo YMD, Tein MS, Lau TK, Haines CJ, Leung TN, Poon PM (1998). Quantitative analysis of fetal DNA in maternal plasma and serum: implications for noninvasive prenatal diagnosis. Am J Hum Genet.

[30] Chiu RWK, Akolekar R, Zheng YWL, Leung TY, Sun H, Chan KCA (2011). Non-invasive prenatal assessment of trisomy 21 by multiplexed maternal plasma DNA sequencing: large scale validity study. BMJ.

[31] Lun FMF, Chiu RWK, Allen Chan KC, Yeung Leung T, Kin Lau T, Dennis Lo YM (2008). Microfluidics digital PCR reveals a higher than expected fraction of fetal DNA in maternal plasma. Clin Chem.

[32] Wang E, Batey A, Struble C, Musci T, Song K, Oliphant A (2013). Gestational age and maternal weight effects on fetal cell-free DNA in maternal plasma. Prenatal Diagnosis.

[33] Rava RP, Srinivasan A, Sehnert AJ, Bianchi DW (2014). Circulating fetal cell-free DNA fractions differ in autosomal aneuploidies and monosomy x. Clin Chem.

[34] Ashoor G, Poon L, Syngelaki A, Mosimann B, Nicolaides KH (2012). Fetal fraction in maternal plasma cell-free DNA at 11-13 weeks’ gestation: effect of maternal and fetal factors. Fetal Diag Ther.

[35] Ashoor G, Syngelaki A, Poon LCY, Rezende JC, Nicolaides KH (2012). Fetal fraction in maternal plasma cell-free DNA at 11-13 weeks’ gestation: relation to maternal and fetal characteristics. Ultrasound in Obstet Gynecol.

[36] Sekizawa A, Yokokawa K, Sugito Y, Iwasaki M, Yukimoto Y, Ichizuka K (2003). Evaluation of bidirectional transfer of plasma DNA through placenta. Hum Genet.

[37] Alberry M, Maddocks D, Jones M, Abdel Hadi M, Abdel-Fattah S, Avent N (2007). Free fetal DNA in maternal plasma in anembryonic pregnancies: Confirmation that the origin is the trophoblast. Prenat Diagn.

[38] Tjoa ML, Cindrova-Davies T, Spasic-Boskovic O, Bianchi DW, Burton GJ (2006). Trophoblastic Oxidative Stress and the Release of Cell-Free Feto-Placental DNA. Am J Pathol.

[39] Wataganara T, Metzenbauer M, Peter I, Johnson KL, Bianchi DW (2005). Placental volume, as measured by 3-dimensional sonography and levels of maternal plasma cell-free fetal DNA. Am J Obstet Gynecol.

[40] Liu FM, Wang XY, Feng X, Wang W, Ye YX, Chen H (2007). Feasibility study of using fetal DNA in maternal plasma for non-invasive prenatal diagnosis. Acta Obstet Gynecol Scand.

[41] Kolialexi A, Tsangaris GTH, Antsaklis A, Mavrou A (2004). Rapid clearance of fetal cells from maternal circulation after delivery. Ann N Y Acad Sci.

[42] Dennis Lo YM, Zhang J, Leung TN, Lau TK, Chang AMZ, Magnus Hjelm N (1999). Rapid clearance of fetal DNA from maternal plasma. Am J Hum Genet.

[43] Kulis M, Heath S, Bibikova M, Queiros AC, Navarro A, Clot G (2012). Epigenomic analysis detects widespread gene-body DNA hypomethylation in chronic lymphocytic leukemia. Nat Genet.

[44] Lister R, Pelizzola M, Dowen RH, Hawkins RD, Hon G, Tonti-Filippini J (2009). Human DNA methylomes at base resolution show widespread epigenomic differences. Nature.

[45] Jiang P, Sun K, Lun FMF, Guo AM, Wang H, Chan KCA (2014). Methy-pipe: an integrated bioinformatics pipeline for whole genome bisulfite sequencing data analysis. PLoS One.

[46] Lun FMF, Chiu RWK, Sun K, Leung TY, Jiang P, Chan KCA (2013). Noninvasive prenatal methylomic analysis by genomewide bisulfite sequencing of maternal plasma DNA. Clin Chem.

[47] Chavan-Gautam P, Sundrani D, Pisal H, Nimbargi V, Mehendale S, Joshi S (2011). Gestation-dependent changes in human placental global DNA methylation levels. Mol Reprod Dev.

[48] Jensen TJ, Kim SK, Zhu Z, Chin C, Gebhard C, Lu T (2015). Whole genome bisulfite sequencing of cell-free DNA and its cellular contributors uncovers placenta hypomethylated domains. Genome Biol.

[49] Kitzman JO, Snyder MW, Ventura M, Lewis AP, Qiu R, Simmons LE (2012). Noninvasive whole-genome sequencing of a human fetus. Sci Transl Med.

[50] Chandrananda D, Thorne NP, Ganesamoorthy D, Bruno DL, Benjamini Y, Speed TP (2014). Investigating and correcting plasma DNA sequencing coverage bias to enhance aneuploidy discovery. PLoS One.

[51] Mailman MD, Feolo M, Jin Y, Kimura M, Tryka K, Bagoutdinov R (2007). The NCBI dbGaP database of genotypes and phenotypes. Nat Genet.

[52] Novoalign software. http://www.novocraft.com.

[53] Li H, Handsaker B, Wysoker A, Fennell T, Ruan J, Homer N (2009). The Sequence Alignment/Map format and SAMtools. Bioinformatics.

[54] Picard Tools. http://broadinstitute.github.io/picard.

[55] McKenna A, Hanna M, Banks E, Sivachenko A, Cibulskis K, Kernytsky A (2010). The genome analysis toolkit: a MapReduce framework for analyzing next-generation DNA sequencing data. Genome Res.

[56] R: A language and environment for statistical computing. R: A Language and Environment for Statistical Computing; 2003.

[57] Jurka J, Kapitonov VV, Pavlicek A, Klonowski P, Kohany O, Walichiewicz J (2005). Repbase update, a database of eukaryotic repetitive elements. Cytogenet Genome Res.

[58] Landt SG, Marinov GK, Kundaje A, Kheradpour P, Pauli F, Batzoglou S (2012). ChIP-seq guidelines and practices of the ENCODE and modENCODE consortia. Genome Res.

[59] Bernstein BE, Birney E, Dunham I, Green ED, Gunter C, ENCODE Project Consortium (2012). An integrated encyclopedia of DNA elements in the human genome. Nature.

[60] ENCODE Project Data Portal. http://genome.ucsc.edu/ENCODE/downloads.html.

[61] Lui YYN, Chik K-W, Chiu RWK, Ho C-Y, Lam CWK, Lo YMD (2002). Predominant hematopoietic origin of cell-free DNA in plasma and serum after sex-mismatched bone marrow transplantation. Clin Chem.

[62] ENCODE Project Cell Types. http://genome.ucsc.edu/ENCODE/cellTypes.html.

[63] Hoffman MM, Ernst J, Wilder SP, Kundaje A, Harris RS, Libbrecht M (2013). Integrative annotation of chromatin elements from ENCODE data. Nucleic Acids Res.

[64] Bailey TL (2011). DREME: motif discovery in transcription factor ChIP-seq data. Bioinformatics.

[65] Smith KR, Bromhead CJ, Hildebrand MS, Shearer AE, Lockhart PJ, Najmabadi H (2011). Reducing the exome search space for Mendelian diseases using genetic linkage analysis of exome genotypes. Genome Biol.

[66] Poptsova MS, Il’icheva IA, Nechipurenko DY, Panchenko LA, Khodikov MV, Oparina NY (2014). Non-random DNA fragmentation in next-generation sequencing. Sci Rep.

[67] Valouev A, Johnson SM, Boyd SD, Smith CL, Fire AZ, Sidow A (2011). Determinants of nucleosome organization in primary human cells. Nature.

[68] Fraser RM, Keszenman-Pereyra D, Simmen MW, Allan J (2009). High-resolution mapping of sequence-directed nucleosome positioning on genomic DNA. J Mol Biol.

[69] Lui YYN, Woo K-S, Wang AYM, Yeung C-K, Li PKT, Chau E (2003). Origin of plasma cell-free DNA after solid organ transplantation. Clin Chem.

[70] Benjamini Y, Speed TP (2012). Summarizing and correcting the GC content bias in high-throughput sequencing. Nucleic Acids Res.

[71] Chan RWY, Jiang P, Peng X, Tam L-S, Liao GJW, Li EKM (2014). Plasma DNA aberrations in systemic lupus erythematosus revealed by genomic and methylomic sequencing. Proc Natl Acad Sci U S A.

