# Supplementary figures and images for: High-resolution characterization of sequence signatures due to non-random cleavage of cell-free DNA
Source: BMC Med Genomics. 2015 Jun 17;8:29. doi: 10.1186/s12920-015-0107-z (PMC4469119; doi:10.1186/s12920-015-0107-z)

Density

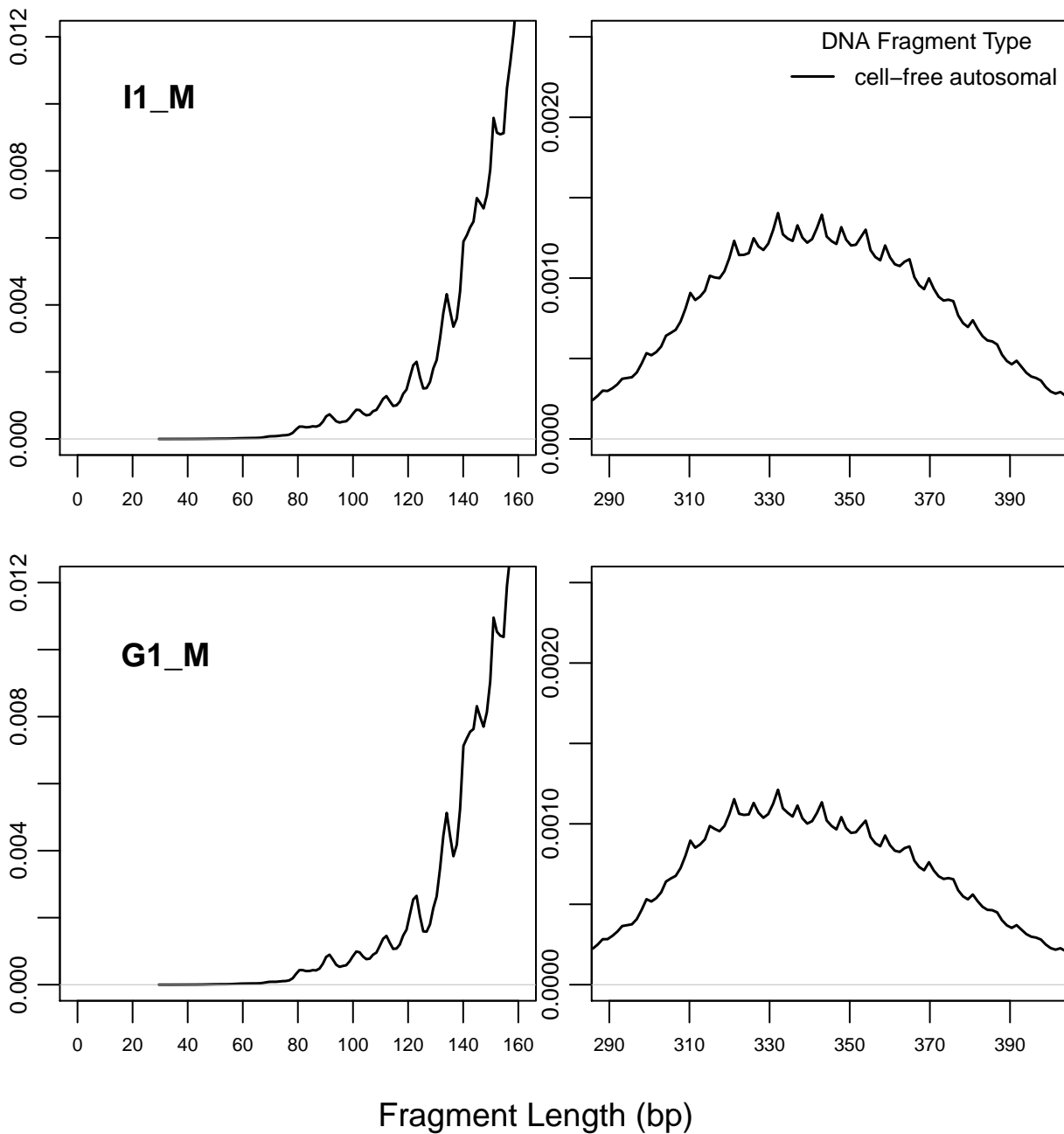

Supplement: Additional file 1: Figure S1. — Size distributions of autosomal cell-free DNA from two subjects (I1_M and G1_M). The plots show the ~ 10 bp periodicity in fragment lengths smaller than 145 bp and the approximate 5 bp periodicity in fragment sizes between 290−390 bp. [file 12920_2015_107_MOESM1_ESM.pdf]

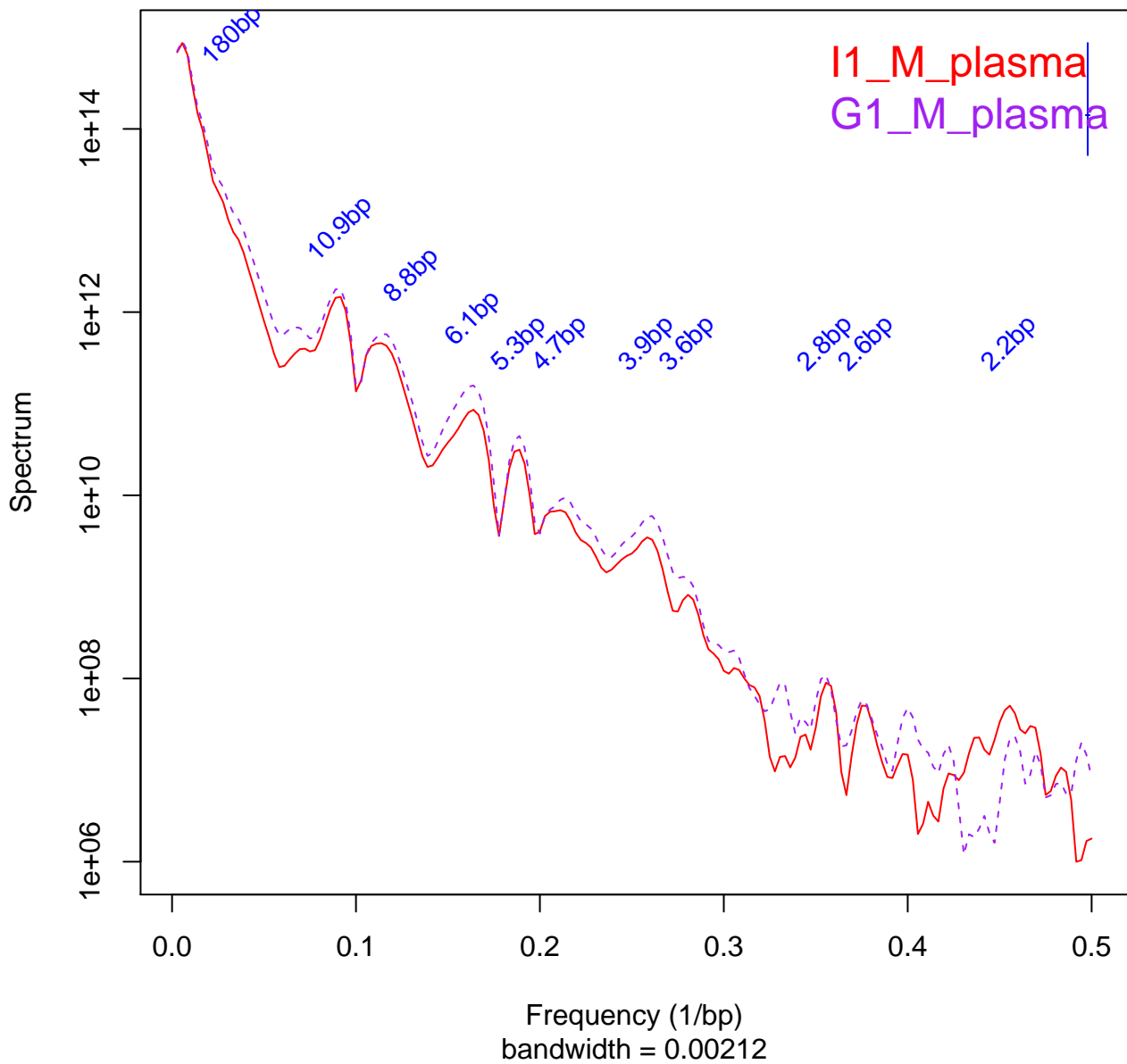

Supplement: Additional file 2: Figure S2. — Smoothed periodogram of the time-series fit to the cell-free autosomal fragment lengths of samples I1_M and G1_M. [file 12920_2015_107_MOESM2_ESM.pdf]

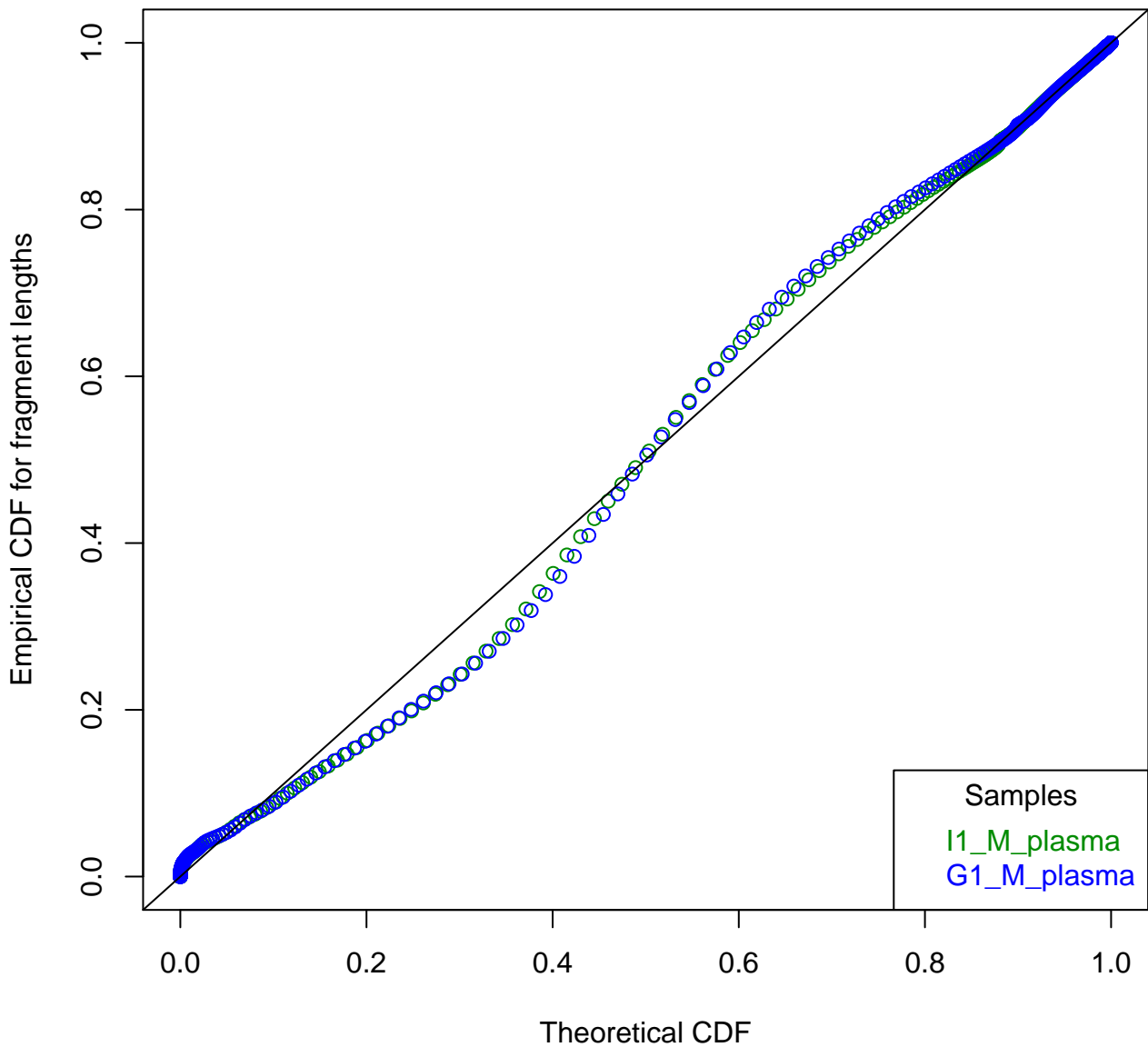

Supplement: Additional file 3: Figure S3. — Goodness of fit of the 3-component Gaussian mixture model fitted for the genome-wide autosomal fragment lengths in cell-free DNA data. While the distribution function of the mixture is the sum of weighted Gaussian probabilities, its inverse is computed numerically. [file 12920_2015_107_MOESM3_ESM.pdf]

# I1\_M\_plasma

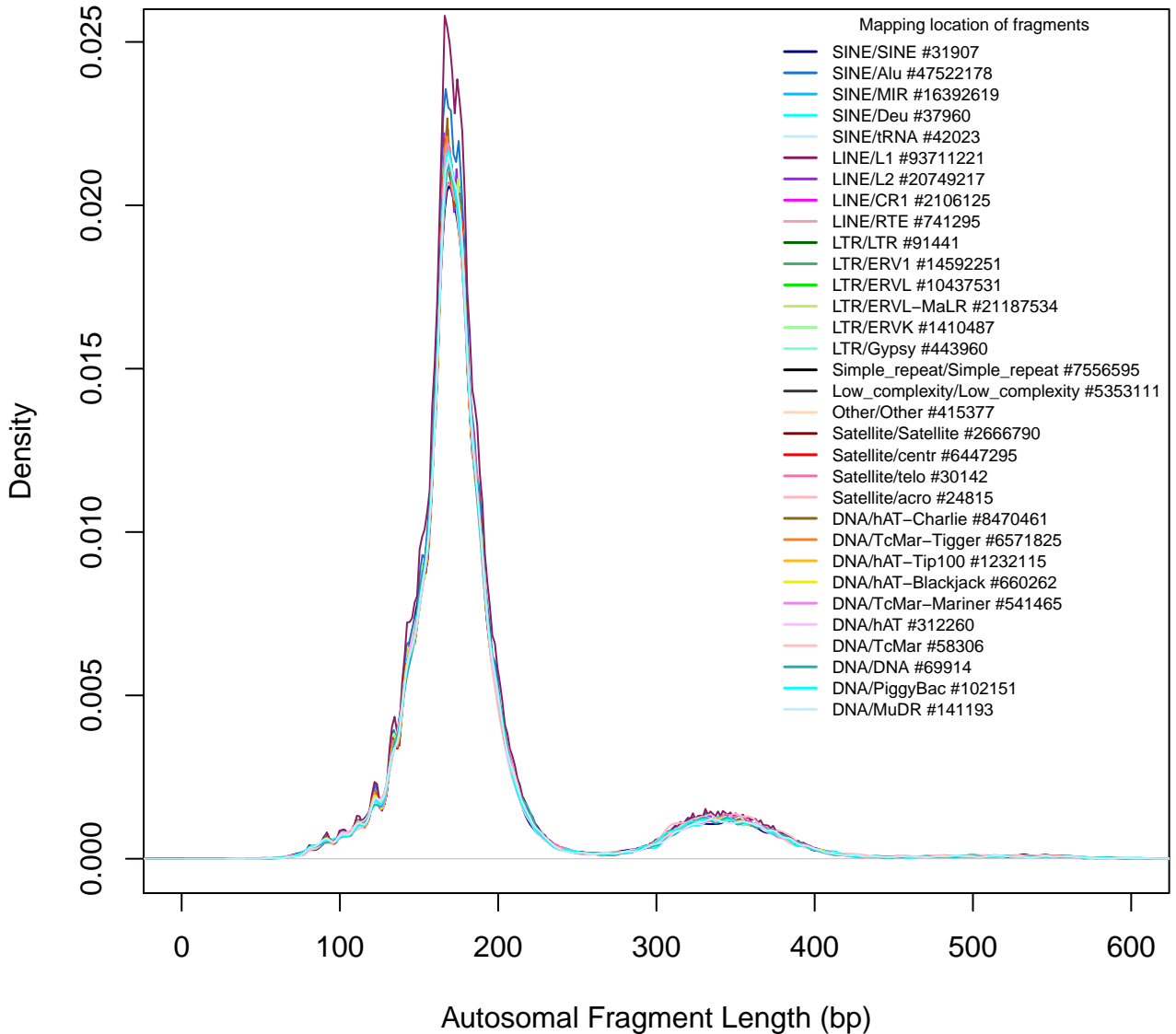

Supplement: Additional file 5: Figure S4. — The density profiles of autosomal fragment lengths originating at 32 different repeat categories in sample I1_M_plasma. The number of fragments used to calculate the size distribution is depicted in the legend beside each repeat category. [file 12920_2015_107_MOESM5_ESM.pdf]

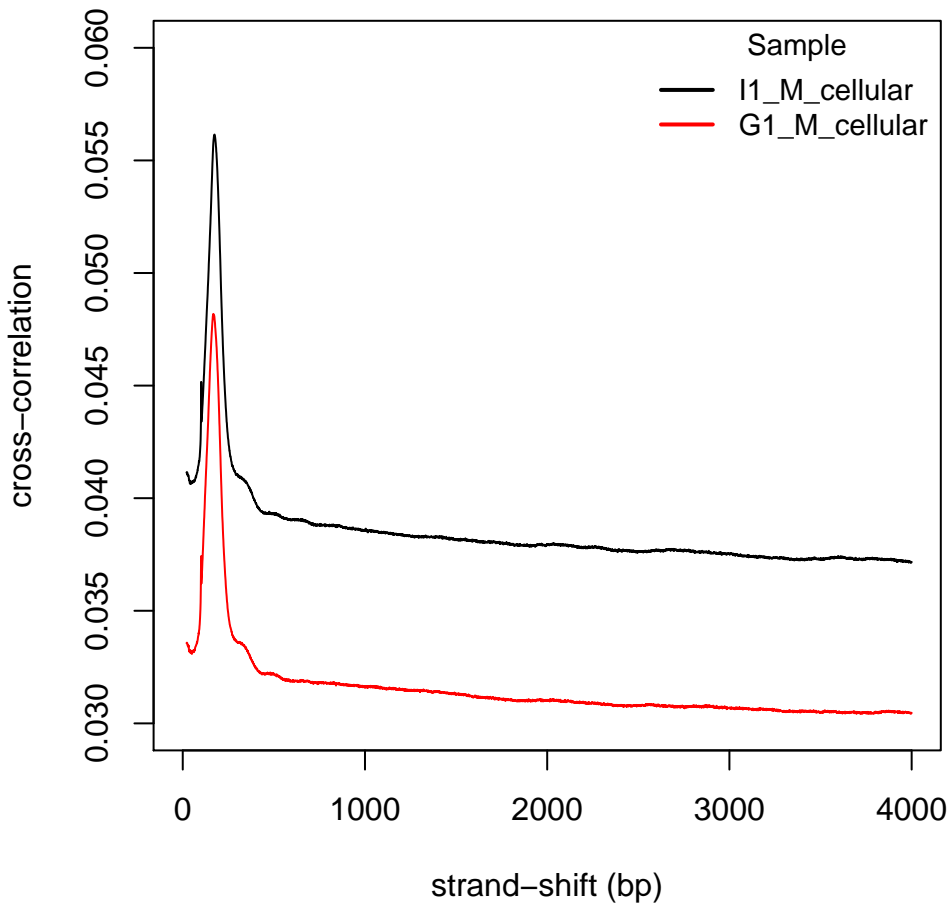

Supplement: Additional file 7: Figure S5. — Strand cross-correlation analysis for cellular DNA. The 3′ strand is shifted with respect to the forward strand in increments of 1 bp and the Pearson’s correlation between the per-position read counts for each strand is calculated to generate this cross-correlation plot. [file 12920_2015_107_MOESM7_ESM.pdf]

Solid line: 100–140 bp (cleavage within nucleosome)

Dashed line: 200–250bp (cleavage within linker)

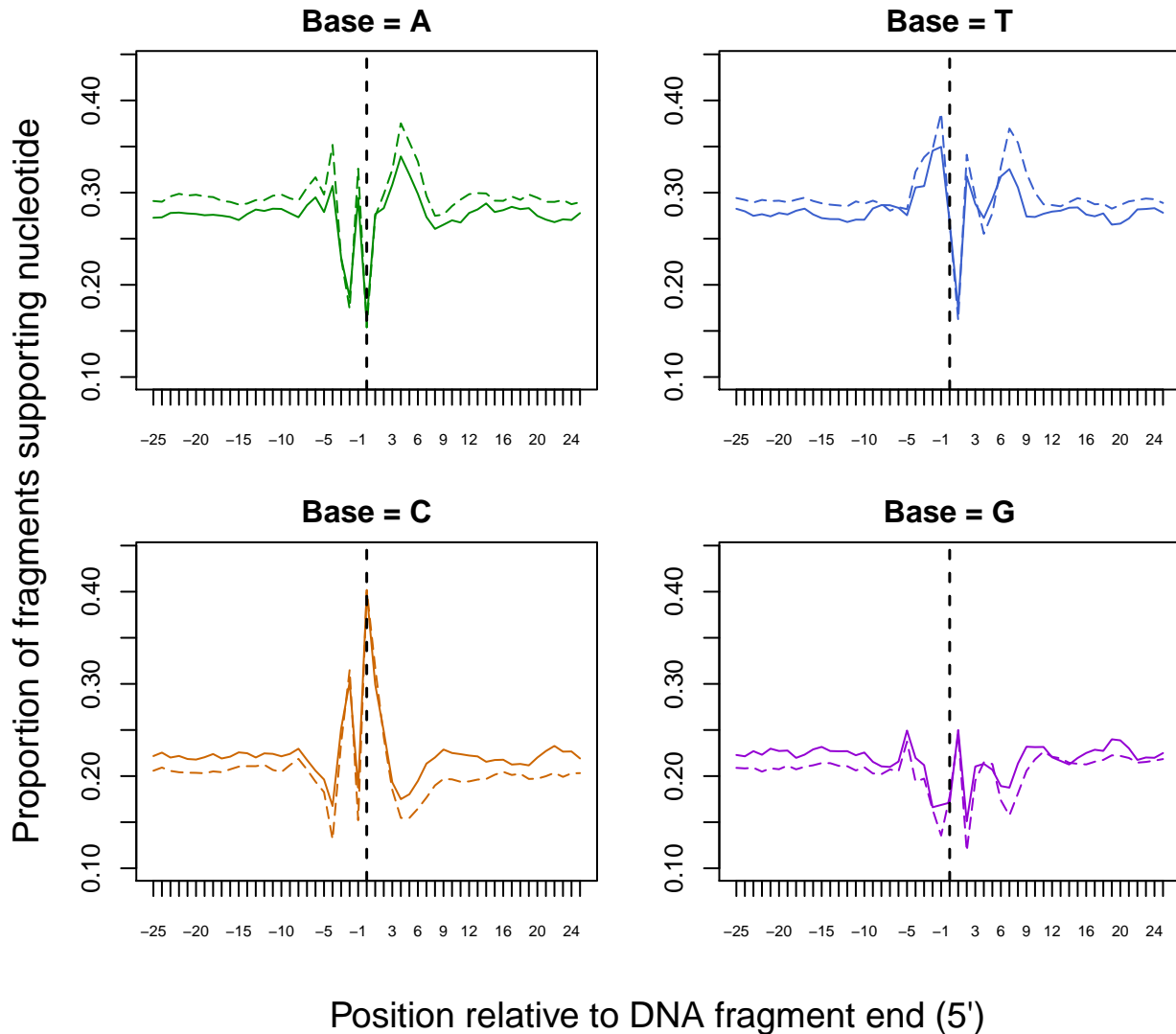

Supplement: Additional file 9: Figure S6. — Mononucleotide frequencies for the region of 51 bp (+/−25 bp) around fragment start sites, separated by fragment length. The y-axis denotes the proportion of each nucleotide at fixed positions relative to the 5′ end of the autosomal DNA fragment and the vertical line at 0 denotes the fragment start. Values are averaged between I1_M and G1_M samples. Fragments are divided into two classes according to their length displayed as solid and dashed lines respectively. [file 12920_2015_107_MOESM9_ESM.pdf]
