# Supplementary material for: High-resolution characterization of sequence signatures due to non-random cleavage of cell-free DNA
Source: BMC Med Genomics. 2015 Jun 17;8:29. doi: 10.1186/s12920-015-0107-z (PMC4469119; doi:10.1186/s12920-015-0107-z)
Supplement: Additional file 10: Figure S7. — Top result from the discriminatory sequence motif analysis using DREME software. [file 12920_2015_107_MOESM10_ESM.pdf]

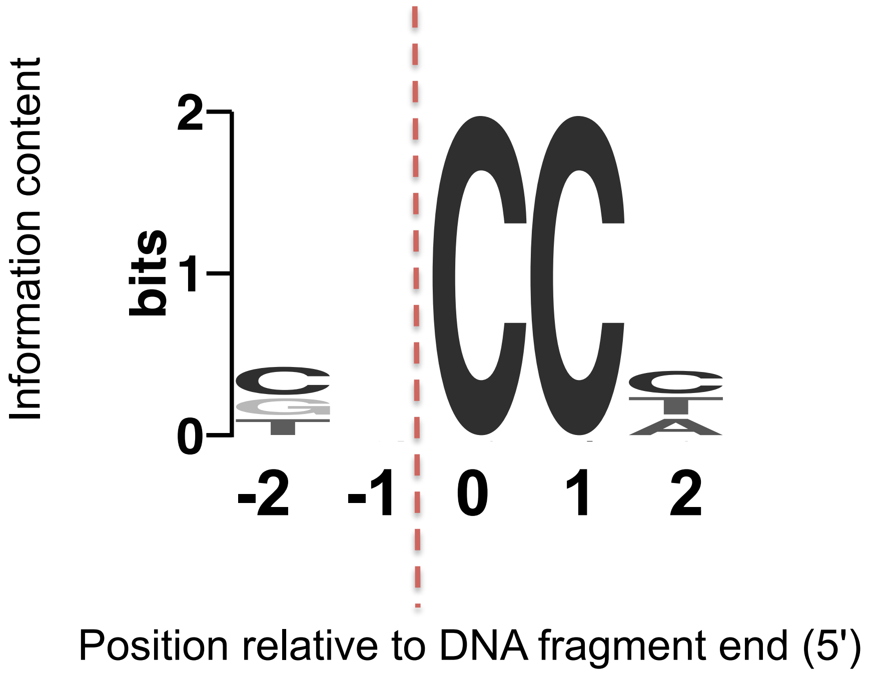

Sample	Number of fragments that support top sequence motif		E-value *
	cell-free DNA	cellular DNA	
I1_M	1421548/10000000	501496/10000000	1.8e-109786
G1_M	1665415/10000000	641746/10000000	5.6e-115017

* The enrichment p-value is calculated using the Fisher's Exact Test for enrichment of the motif in cell-free DNA compared to cellular sequences.
